# Edge-Sensitive Left Ventricle Segmentation Using Deep Reinforcement Learning

**DOI:** 10.3390/s21072375

**Published:** 2021-03-29

**Authors:** Jingjing Xiong, Lai-Man Po, Kwok Wai Cheung, Pengfei Xian, Yuzhi Zhao, Yasar Abbas Ur Rehman, Yujia Zhang

**Affiliations:** 1 Department of Electrical Engineering, City University of Hong Kong, Tat Chee Avenue, Kowloon, Hong Kong, China; eelmpo@cityu.edu.hk (L.-M.P.); xian.pf@my.cityu.edu.hk (P.X.); yzzhao2-c@my.cityu.edu.hk (Y.Z.); yzhang2383-c@my.cityu.edu.hk (Y.Z.); 2 School of Communication, The Hang Seng University of Hong Kong, Hang Shin Link, Siu Lek Yuen, Shatin, Hong Kong, China; keithcheung@hsu.edu.hk; 3 TCL Corporate Research (HK) Co., Ltd., 22 Science Park East Avenue, Shatin, Hong Kong, China; yasir.abbas42@gmail.com

**Keywords:** left ventricle segmentation, image segmentation, deep reinforcement learning, double deep Q-network, Markov decision process

## Abstract

Deep reinforcement learning (DRL) has been utilized in numerous computer vision tasks, such as object detection, autonomous driving, etc. However, relatively few DRL methods have been proposed in the area of image segmentation, particularly in left ventricle segmentation. Reinforcement learning-based methods in earlier works often rely on learning proper thresholds to perform segmentation, and the segmentation results are inaccurate due to the sensitivity of the threshold. To tackle this problem, a novel DRL agent is designed to imitate the human process to perform LV segmentation. For this purpose, we formulate the segmentation problem as a Markov decision process and innovatively optimize it through DRL. The proposed DRL agent consists of two neural networks, i.e., First-P-Net and Next-P-Net. The First-P-Net locates the initial edge point, and the Next-P-Net locates the remaining edge points successively and ultimately obtains a closed segmentation result. The experimental results show that the proposed model has outperformed the previous reinforcement learning methods and achieved comparable performances compared with deep learning baselines on two widely used LV endocardium segmentation datasets, namely Automated Cardiac Diagnosis Challenge (ACDC) 2017 dataset, and Sunnybrook 2009 dataset. Moreover, the proposed model achieves higher *F-measure* accuracy compared with deep learning methods when training with a very limited number of samples.

## 1. Introduction

The movement of the boundary of the left ventricle (LV) could be used to measure mechanical dyssynchrony. For the diagnosis and treatment of heart diseases, doctors usually need to delineate the LV boundary from the cardiac magnetic resonance (MR) images manually, which is time-consuming and reduces the efficiency of diagnosis. Hence, automatic and robust identification of the LV boundary is important. However, due to the motion of LV during image acquisition, the captured images are usually characterized by blurring and intensity inhomogeneity, which makes the segmentation of magnetic resonance LV images challenging. To address this problem, numerous methods have been proposed for the automatic segmentation of LV images, which can be divided into three groups: hand-engineered features-based segmentation, deep learning-based segmentation, and reinforcement learning based segmentation.

Traditionally, segmentation was carried out using hand-crafted features like threshold or edge/region information to obtain the final segmentation results. For instance, Rundo et al. [1] exploited the split-and-merge algorithm to find multiple homogeneous seed-regions and performed the segmentation through the multi-seed region growing procedure guided by appropriate similarity properties of intensity features. However, poor image quality may limit the performance of these methods. In contrast, the deep learning-based LV segmentation methods have achieved state-of-the-art performance [2,3,4,5]. It extracts features from the input image in a hierarchical fashion, i.e., from low-level features to more abstract and data specific features. Shallow layers in the neural networks have a narrow receptive field, which can learn features from the local area while deep layers have a larger receptive field, which can learn abstract features like semantic information. However, deep learning approaches are data-driven and their performances are highly correlated with the amount of data available for training. LV dataset is relatively small and hard to acquire, which restricts the performance of deep learning-based approaches.

Reinforcement learning (RL) has achieved considerable attention since Alpha Go defeated the human champion on Go Game. It is an interactive process between a software agent or multiple software agents and the environment. The environment gives the agent an initial state, and the agent interacts with the environment by making an action, receiving an immediate reward, and goes to the next state. The goal of the artificial agent is to learn an optimal policy to maximize the accumulated reward [6,7]. Deep reinforcement learning (DRL) employs concepts and principles in RL and builds a neural network to represent a value function or a policy. The most basic and widely used method in DRL is Deep Q-Network (DQN) [8] proposed by Mnih et al. in 2012. DQN is the first algorithm that combines reinforcement learning and deep learning. It achieved human-level performance in Atari games and even beyond human players’ performances in some games. Since then, more and more researches on DQN have been carried out. Techniques like double DQN [9], dueling DQN [10], recurrent DQN [11] and prioritized experience replay [12] have been proposed to improve the performance of vanilla DQN.

DRL has been widely researched in object detection [13,14,15], video tracking [16], intelligent driving [17], etc. However, in the field of image segmentation, especially medical image segmentation, DRL methods are quite rare. Inspired by the human segmentation process, this paper explores how to utilize DRL to perform LV segmentation. For instance, a person can complete the task of delineating an object from an image by firstly finding an initial point on the edge of the object, and then gradually positioning other edge points. If we treat the LV segmentation process as deciding the LV edge points one by one, then the DRL agent can imitate the human process to perform image segmentation. The initial state of the agent is given when finding the first edge point and the action is to draw the next LV edge point. After making the action, the agent will receive a new state. This process is repeated until the agent draws a closed LV contour. The DRL agent records the coordinates of all positioned boundary points to obtain a closed contour, which is fundamentally different from the deep learning method that outputs the segmentation probability of each pixel.

**Figure 1:**
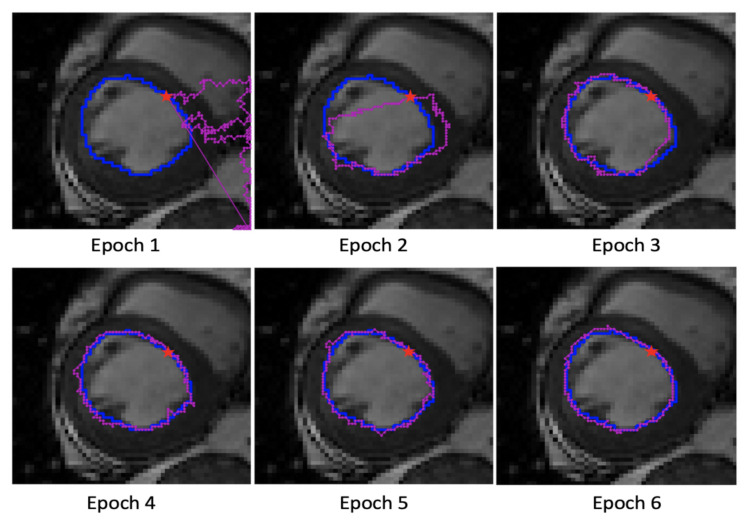
An example of the segmentation results of an image on Automated Cardiac Diagnosis Challenge (ACDC) 2017 training dataset across different epochs in the training process. The ground truth (GT) boundary is plotted in blue and the magenta dots are the points found by Next-P-Net. The red pentagram represents the first edge point found by First-P-Net.

An example of the segmentation performance of the proposed method among different training epochs is shown in Figure 1. The model first finds the initial edge point and iteratively locates the next edge points coordinates, which corresponds closely to the field of vision and viewing patterns of the human eye. The localized edge points constitute the final image segmentation result. The key module of the proposed method is the DRL agent that learns how to find the next edge point step by step. In this paper, we choose Double DQN as the core algorithm because it reduces the overestimation of vanilla DQN. This significantly contributes to the stability of learning. The main contributions of this paper are as follows:

(1) We formulate the segmentation problem as a sequential decision-making process (Markov Decision Process), optimize it through DRL using double Deep Q-Network and innovatively define state, action, and reward.

(2) The proposed model records a list of the boundary points coordinates, which is fundamentally different from conventional deep learning-based LV segmentation methods that output the segmentation probability of each pixel.

(3) The segmentation process of the proposed model corresponds closely to the field of vision and viewing patterns of the human eye.

(4) The proposed model can address the problem that deep learning-based methods require big data because it can be trained by utilizing a very limited number of samples and achieves higher *F-measure* accuracy compared with deep learning (DL) baseline methods.

The remainder of this paper is organized as follows. In Section 2, we briefly review related works in image segmentation. In Section 3, we provide the methodology of the proposed DRL method for LV segmentation in detail. Later, the experimental results and discussions are elaborated in Section 4. Finally, we conclude the paper and explore avenues for further research of our work in Section 5.

## 2. Related Works

**Deep learning-based medical image segmentation.** Deep learning methods have achieved great success in image segmentation since its successful incarnation in 2012. Minaee et al. [18] provided a comprehensive review of the deep learning techniques in image segmentation. Chen et al. [19] comprehensively reviewed the recent deep learning techniques for cardiac image segmentation, and Litjens et al. [20] provided a thorough overview of state-of-the-art deep learning approaches in cardiovascular image analysis. For the left ventricle segmentation, top performing methods are based on deep learning technologies [21,22,23,24,25], in particular fully convolutional networks (FCN) [26] and U-Net [2]. Both of them can be applied to the entire input image and perform convolution/deconvolution to output a semantic likelihood map. Abdeltawaba et al. [27] proposed a novel FCN2 architecture based on FCN to reduce the memory footprint for cardiac segmentation. Recently, U-Net architecture is the most well-known deep learning architecture in medical image segmentation. Liu et al. [28] provided a comprehensive literature review of U-shaped networks applied to medical image segmentation tasks. Several improvements have been made to U-Net architecture, e.g., UNet++ [21], Attention U-Net [22]. UNet++ re-designed the skip pathways that connect the two sub-networks in UNet. Attention U-Net introduces a novel attention gate to highlight salient features that are passed through the skip connections. Rundo et al. [29] incorporated Squeeze-and-Excitation blocks [30] into every encoder or decoder block in U-Net to boost the segmentation performance with feature recalibration. Galea et al. [31] presented a practical approach to perform cardiac image segmentation through ensembling of DeepLab-V3+ [32] and U-Net [2].

Different from the classic segmentation method proposed by Militello et al. [33], who introduced a user-friendly Graphical User Interface tool to support radiologists in epicardial adipose tissue (EAT) segmentation and quantification, Commandeur et al. [34] exploited a convolutional neural network (CNN) for automated and fast EAT volume and density quantification. Moreno et al. [35] employed two CNNs to develop a fully automatic LV segmentation method. The first one defined the region of interest of the cardiac chambers and the second one, which is based on U-Net, segmented the myocardium and the blood pool. Romaguera et al. [36] and Nasr-Esfahani et al. [37] proposed a deep fully convolutional neural network architecture to perform LV segmentation. Some authors have combined deep learning with classical segmentation method, where the most common combination was deep learning and deformable model [38,39,40]. Avendi et al. [38] combined deep learning and deformable models to conduct fully automatic LV segmentation, where deep learning algorithms detect and infer the shape of LV. In [39], Ngo et al. integrated a deep belief network into a level-set model, where the deep belief network can reliably model the shape and appearance of the LV.

**Reinforcement learning-based image segmentation.** Apart from deep learning methods, various studies have been carried out on reinforcement learning-based image segmentation methods. Shokri et al. [41] introduced a simple Q learning model to find the optimal threshold for digital images. Song et al. [42] introduced an automatic seed generation technique with dueling DQN to solve the interactive segmentation problem. The function of this reinforcement learning networks is to find several seeds to distinguish foreground and background, and the segmentation process is based on the random walker segmentation algorithm [43]. Han et al. [44] made the assumption that object segmentation mainly relies on the interaction between object regions and their context, and implemented a cutting-policy network (CPN) to find the optimal object box and context box, and a cutting-execution network (CEN) to carry out object segmentation. The CPN is a Deep Q Network and the CEN takes the architecture of FC-DenseNet [45]. The reinforcement learning network in these methods cannot complete the object segmentation task directly since they barely provide useful information to the segmentation part, and the segmentation process is achieved via other networks. In [46,47], the authors constructed a recurrent neural network to model the sequence of 2D vertices of the polygon outlining an object and reformulate the polygon prediction task as a RL problem.

**Reinforcement learning-based medical image segmentation.** RL architectures have also been applied in analyzing medical images obtained from magnetic resonance imaging (MRI), computerized tomography (CT) scan, ultrasound (UlS), etc. However, to the best of our knowledge, the research on medical image segmentation is limited [48,49,50,51,52,53,54], especially in LV segmentation [55,56,57]. In [58], Mahmud et al. reviewed various important applications of deep learning and reinforcement learning to biological data. RL methods tended to segment the medical images by exploiting a suitable threshold earlier, and adopted the simple thresholding method for segmentation. As a result, the final segmentation output was rough and inaccurate. For instance, the methods proposed in [52,53] divided the prostate ultrasound image into several sub-images and employed a Q learning scheme to optimally find the appropriate local threshold and structuring element values for each sub-image individually. In 2019, Liao et al. [54] employed an Actor-Critic model to iteratively refine the coarse previous segmentation by integrating user interactions in order to get a precise result for 3D medical image segmentation. However, this method requires an initial segmentation result and human hints based on error regions. Mortazi et al. [57] developed a network optimization search algorithm based on a policy gradient (PG) reinforcement learning. In this paper, we show that DRL enables us to implement LV segmentation in a more human-like manner. It can accomplish the image segmentation process directly and achieve acceptable performance after several epochs.

## 3. Methodology

**Figure 2:**
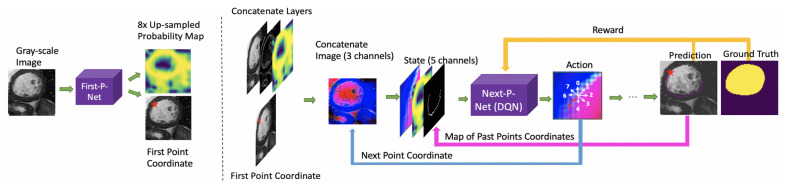
The overall process of the proposed system: The First-P-Net finds the first edge point and generates a probability map of edge points positions. The Next-P-Net locates the next point based on the previous edge point and image information.

The overall process of the proposed system is shown in Figure 2. The proposed system consists of two neural networks: First-P-Net and Next-P-Net.

**First-P-Net**: The goal of First-P-Net is to find a good initial (or first) edge point to start the segmentation process and generate the probability feature map of edge point positions. First-P-Net is a traditional CNN network, which is trained before Next-P-Net.

**Next-P-Net**: After obtaining the first edge point from First-P-Net, Next-P-Net generates the next point based on the previous edge points and image information. The system proceeds iteratively with the image and edge points. The input of Next-P-Net is an edge point-centric concatenated matrix. In addition to the original grayscale image, the concatenated input contains the edge information obtained by Sobel filter, two probability feature maps of edge points positions and a map of past points. Given the concatenated matrix as input, the output of Next-P-Net is the coordinate of the next edge point. Segmentation of the image by adopting all localized points in the current step results in a new binary mask, which can be utilized for computing the reward signal by comparing it with the ground truth mask. The reward signal evaluates the goodness or badness of the next point generated by Next-P-Net and is employed to update the Next-P-Net parameters.

The cyclic operations for finding the next edge point are repeated with the reward signal updating the DRL neural network in the training process. In the testing process, the system runs without the reward part, and generates new point iteratively until reaching the end point. We obtain the final segmentation binary mask by plotting all points localized by First-P-Net and Next-P-Net.

### 3.1. Markov Decision Process (MDP)

The fundamental part of the proposed system is the optimal generation of the next edge point. Generating acceptable edge points can be formalized as MDP because given the current state and operation, finding the next edge point is independent of the past. A MDP is a tuple <S,A,P,R,γ>, where *S* is a set of states, *A* is a set of chosen actions, *P* is the state transition probability matrix, *R* is a set of immediate rewards and γ is a discount factor in the range [0, 1]. In our proposed segmentation system, the state, action and reward of MDP are defined as follows.

**State**: The state is the input of Next-P-Net, which contains important image information to enable the DRL agent to make the best action. In addition to the original grayscale image (size: W×H), some auxiliary knowledge should be added to provide more details. Since our method is an edge-based method, we detect the edge information using a Sobel operator. Besides, the size of the original probability map of edge points positions generated by First-P-Net is one-eighth of the input height and width. Then, the 8× up-sampled version of the probability map, which has the same size as that of the input image, is also concatenated. Therefore, the concatenated image includes three layers, which are grayscale layer, edge information layer (Sobel layer) and 8× up-sampled probability map layer.

In our problem formulation, an edge point-centric cropped matrix is defined as the state. The edge point is given by the first edge point found by First-P-Net or the previous point found by Next-P-Net. Each state consists of 3 layers cropped from the concatenated image (size: w×h×3) and two other layers (size: w×h×2). One of the additional layers is the global probability map of edge points positions, which contains important global information. The original probability map generated by the First-P-Net is resized to w×h to form the global probability map. Another additional layer is cropped from the map of previous edge points coordinates. The trajectory of previously identified points records the interaction between the DQN agent and the environment. The trajectory history allows the agent to remember the positions of previous identified edge points and enables the agent to make more coherent actions. The default value of the past points map is set to 0 because no point was found at the beginning. Thereafter, this map is updated by adding the newly localized point and filling the point position with a value of 1.

In summary, the state has five layers (size: w×h×5). Four of them are cropped from the concatenated image or past points’ map. The fifth layer is the fixed global probability map. Regardless of the position of the edge points, the spatial dimensions of the state (w×h) are fixed and should be chosen appropriately according to the image size. w≪W and h≪H since firstly, if the cropped size is too small, it cannot provide enough information to Next-P-Net and may result in terrible action decision. On the other hand, we implement double DQN [9] as the reinforcement learning algorithm. Q-learning based methods need to store a replay buffer which contains a certain number of state transitions. If the cropped size is too large, the replay buffer will occupy enormous computer resources. In addition, as the system proceeds by finding the edge points iteratively, it is unnecessary to exploit large image grayscale information. The idea finely accords with the human segmentation process by modeling the segmentation task as a sequential decision-making process. When human segments an object from the background, the person mainly focuses on the local information and combining global information to some extent. In the proposed model, the cropped layers in the state provide the local information and the global probability map provides the global information. When the size exceeds the original image, a crop and resize function like that in Fast R-CNN [59] is employed to make sure that the size of the cropped image is exactly w×h.

**Figure 3:**
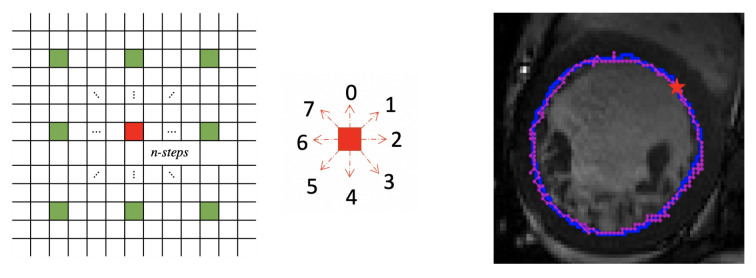
The left image defines the nskipneighborhoods centered on the red point. The green points represent the eight skip neighborhoods of the red point. The middle image shows the defined action space and the corresponding action directions. The right image gives an example of the segmentation result. The ground truth (GT) boundary is plotted in blue and the magenta dots are the points found by Next-P-Net. The red pentagram represents the initial edge point.

**Action**: Given the state, the DQN agent outputs one action from the action space. Centered on the edge point, eight actions corresponding to the eight “neighborhoods” of the edge point are determined. The “neighborhoods” are defined as n-pixels distance from the edge point. Figure 3 illustrates the defined neighborhoods of a given point, where we term *n skip neighborhoods*. The action space in step *t* is defined as $A_{t}=\left\{0,1,2,3,4,5,6,7\right\}$. The different number represents different action for the corresponding neighborhood chosen. The grids in the left image represent the image pixels. Suppose the agent has located the red edge point, the action space is defined as the 8 skip neighborhoods centered on the located point. The agent locates the next corresponding edge point according to the action value output. For instance, if the action is “4”, the next edge point is the green pixel under the red pixel. If the action is “7”, the next edge point is the green pixel in the upper right corner of the red pixel. The traditional definition of eight neighborhoods only allows the agent to move one step at each time. Thus, it takes a lot of steps for the agent to complete a closed contour. Using skip neighborhoods helps the agent to segment an image more effectively.

**Reward**: The immediate reward signal evaluates the goodness or badness of the action generated by the DQN agent. It is the interaction result between the agent and the environment. If the agent finds the next edge point optimally, it should be given a high immediate reward. Otherwise, the immediate reward should be small. The goal of a RL system is to maximize the cumulative reward. Generally, the reward is related to the evaluation metrics. For our problem formulation, one of the most intuitive evaluation metrics in LV segmentation is *F-measure*. A basic reward function regarding *F-measure* is to employ the difference of *F-measure* between the current segmentation mask and the previous segmentation mask. If the difference is greater than 0, the reward should be positive since the current action selected by the agent is acceptable and it makes the *F-measure* become larger than the previous one. However, we found that using the difference in Intersection over Union (*IoU*) achieves better segmentation results than using difference *F-measure* as difference *IoU* gives a more accurate evaluation on the current action and it can effectively avoid the clustering of points. Hence, we define difference IoU reward as $R_{diff\_IoU}$.

Meanwhile, the distance between the localized edge point and the ground truth point can also evaluate the effectiveness of the DQN agent. If the distance is smaller than 10, the agent will obtain a positive $R_{edge\_dist}$ reward which is inversely proportional to the distance. However, if the distance exceeds 10, the reward is 0. Hence, we define two reward functions according to difference *IoU* and edge distance as $R_{diff\_IoU}$ and $R_{edge\_dist}$.

The definition of $R_{diff\_IoU}$ is as follows: $R_{diff\_IoU}=$

(1) $$\left\{\begin{array}{cc} 1, & IoU\left(M_{curr},GT\right)-IoU\left(M_{prev},GT\right)>0\\ 0, & IoU\left(M_{curr},GT\right)-IoU\left(M_{prev},GT\right)=0\\ -1, & IoU\left(M_{curr},GT\right)-IoU\left(M_{prev},GT\right)<0 \end{array}\right.$$

where $M_{curr}$ is the segmentation binary mask computed by the first edge pixel and all localized next points. $M_{prev}$ is the segmentation binary mask which is computed by the first edge pixel and all localized next points except for the last 5 points. GT is the ground truth mask. IoU(x,y) is the IoU between *x* and *y*. We employ the interval of 5 points to compute difference *IoU* reward since it helps the stability of the training process comparing with using the interval of 1 point. With a 1-point interval, the difference *IoU* between the current segmentation mask and previous mask segmented one step earlier is too small and unable to evaluate the current action.

The definition of $R_{edge\_dist}$ is as follows:

(2) $$R_{edge\_dist}=\left\{\begin{array}{cc} ratio*(10-dist), & dist<10\\ 0, & otherwise \end{array}\right.$$

where dist donates the minimum distance between the localized edge point and the ground truth point. The ratio is set to 0.05 in our system. The introduction of the ratio factor is to normalize edge distance reward to [0, 0.5] so that the difference *IoU* reward remains to be the dominant and most direct reward in our model. Otherwise, if the edge distance reward accounts for a large portion of the total reward, the points found by Next-P-Net may gather at the first point and the agent will always get high edge distance reward regardless of smaller difference *IoU* reward. Thus, normalizing the edge distance reward to a small interval highly encourages the agent to find edge points which increase difference *IoU*.

In the experiments, we found that some gathering situations still occurred even though we have normalized the edge distance reward. To discourage the occurrence of such situations, we define points clustering reward as

(3) $$R_{points\_clus}=\left\{\begin{array}{cc} -0.5, & std<10\\ 0, & otherwise \end{array}\right.$$

where std is the standard deviation of the last 20 edge points coordinates. Adding this reward positively helps the agent to avoid turning around one edge point and accelerates the convergence of the proposed model. The final immediate reward *r* is the sum of the above rewards and is given by Equation (4).

(4) $$r=R_{diff\_IoU}+R_{edge\_dist}+R_{points\_clus}$$

**Figure 4:**
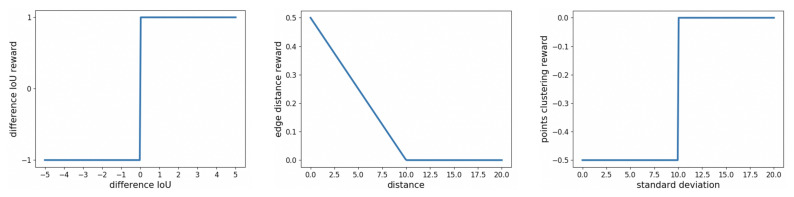
Three separate reward functions: difference *IoU* reward function, edge distance reward function and points clustering reward function.

According to the ranges of three separate rewards, the range of immediate reward *r* is [−1.5, 1.5]. The three separate reward functions are plotted in Figure 4.

### 3.2. Double Deep Q-Network (Double DQN)

We implemented double DQN [9] to train the DRL agent. Double DQN learns the action-value function Q(s,a) through a deep neural network. Q(s,a) is the expected cumulative reward starting from state *s*, taking action *a*. The optimal policy can be obtained from maximizing *Q* function:

(5) $$a=\underset{a\in A}{\arg\max}\;Q\left(s,a\right)$$

Double DQN utilizes two neural networks of the same structure: evaluation network and target network. The evaluation network parameters are updated in each iteration while the target network parameters are fixed during a certain number of iterations. After several iterations, the parameters of evaluation network are assigned to the target network. This fixed target network method has significantly improved reinforcement learning stability. Besides, ϵ-greedy policy is employed to balance exploration and exploitation. ϵ-greedy policy is an exploration policy with a probability of ϵ for the agent to randomly select an action and a probability of 1-ϵ for the agent to select the action that maximizes the Q function. In addition, Q learning method is an off-policy method, so the system stores state transitions $\left(s,a,r,s^{{}^{\prime }}\right)$ to a replay memory unit, where $s^{{}^{\prime }}$ is the next state, and randomly selects some experiences from this memory unit. This experience replay disrupts the correlation between experiences and makes neural networks update more efficiently. The target *Q* value is expressed as:

(6) $$Q_{targ}=r+\gamma Q\left(s^{{}^{\prime }},\underset{a^{{}^{\prime }}}{\arg\max}\;Q\left(s^{{}^{\prime }},a^{{}^{\prime }};\theta_{eval}\right);\theta_{targ}\right)$$

where $a^{{}^{\prime }}$ is the action of next state $s^{{}^{\prime }}$, $\theta_{eval}$ is the evaluation network parameters and $\theta_{targ}$ is the target network parameters. The loss function for training the double DQN can be expressed as:

(7) $$Loss\left(\theta_{eval}\right)={\left(Q_{targ}-Q\left(s,a;\theta_{eval}\right)\right)}^{2}$$

### 3.3. Model Architecture

**Figure 5:**
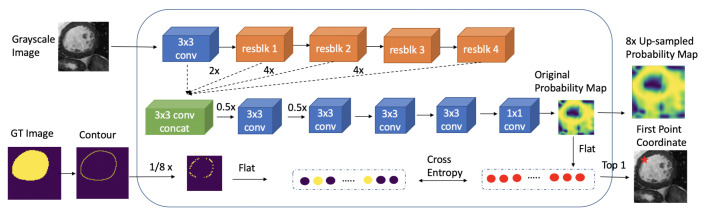
The architecture of First-P-Net. 3 × 3 conv or 1 × 1 conv: 3 × 3 or 1 × 1 convolution layer followed by batch normalization and ReLU activation function. resblk: revised ResNet Block. 2×: 2 upsampling. 4×: 4 upsampling. 0.5×: 0.5 downsampling. 1/8×: 1/8 downsampling.

**Figure 6:**
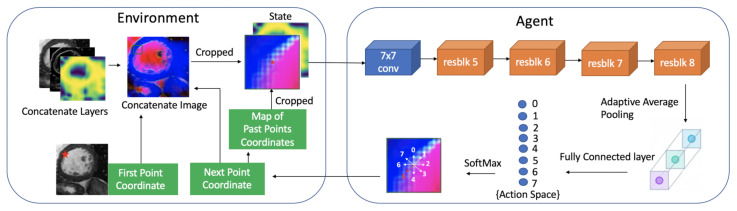
The architecture of Next-P-Net. 7 × 7 conv: 7 × 7 convolution layer followed by batch normalization and ReLU activation function. resblk: ResNet Block.

The architectures of First-P-Net and Next-P-Net are illustrated in Figure 5 and Figure 6. The input sizes of First-P-Net and Next-P-Net are [W×H×3]:[368×368×3] and [w×h×5]:[51×51×5], respectively and the inputs are normalized to [−1, 1]. The backbones of First-P-Net and Next-P-Net are ResNet-50 and ResNet-18 [60]. To decrease the complexity of neural networks, we modify ResNet architecture by reducing the number of layers used in the network. In First-P-Net, we modify ResNet-50 architecture by reducing the number of layers to layers=[2,2,2,2]. Since the spatial dimensions of different block outputs of ResNet are not equivalent, they cannot be concatenated directly. We employ 3×3 convolution and bilinear up-sampling with corresponding scale factor to the block outputs followed by batch normalization [61] and ReLU non-linearities to resize their dimensions into 184×184×64. Then these four blocks are concatenated to 184×184×256. After obtaining the concatenated features, we add four convolutional blocks with a kernel size of 3×3 to output a feature map with size 46×46×16. The probability map is obtained by employing 1×1 convolution to the feature map. Afterward, the probability map is flattened and the log SoftMax function is employed to select the top 1 value as the predicted first edge point. Next-P-Net is a double DQN. We also modify ResNet-18 architecture by using layers=[1,1,1,1] to decrease the complexity. After that, an adaptive average pooling and a fully connected layer are performed on the ResNet feature map to output the action probability.

## 4. Experiments

To demonstrate the efficiency of the proposed model, we performed experiments on two widely used public LV segmentation datasets: ACDC 2017 dataset [62], Sunnybrook 2009 dataset [63], and a set of 15 subjects randomly selected from the ACDC dataset. In addition, we also analyze the effect of different states and rewards on the proposed model.

### 4.1. Datasets

**Automated Cardiac Diagnosis Challenge 2017**-ACDC 2017 dataset [62]. This dataset is composed of 100 sequences from 20 healthy subjects and 80 diseased subjects. These diseased subjects can be divided into four classes: heart failure with infarction, dilated cardiomyopathy, hypertrophic cardiomyopathy, abnormal right ventricle. The ground truth segmentations of LV endocardium and epicardium references are only available for both end-diastolic and end-systolic phase instances by one clinical expert. This dataset can also be utilized to classify different sequences into the corresponding type of disease. In this work, our goal is to segment LV endocardium. Since the LV covers only a small region in cardiac MRI, the RoI around the LV is extracted. A total of 1670 RoIs on this dataset are extracted and images are resized to 368×368 with bilinear interpolation method.

**The Sunnybrook Cardiac MR Left Ventricle Segmentation Challenge**-Sunnybrook 2009 dataset [63]. Cardiac images in this dataset are acquired from 45 sequences, comprising heart failure with ischemia, heart failure without ischemia, hypertrophic cardiomyopathy and normal subjects. It is an early published dataset for automated myocardium segmentation from short-axis MRI, held by a MICCAI workshop in 2009. For each patient record, the hand-drawn contours of the left endocardium and epicardium coordinates for End-Diastolic (ED) and End-Systolic (ES) slices are given as segmentation ground truth. The contours are saved in text files that consist of the x-coordinates and y-coordinates of contour points. A total of 803 RoIs on this dataset is extracted and images are resized to 368×368 with bilinear interpolation method.

### 4.2. Training

Both networks are trained from scratch. Neither image pre-processing method nor data augmentation is adopted in the training process. First-P-Net is similar to the residual encoder architecture of [47]. The skip connections in First-P-Net combine low-level features like edges and high-level features like semantic information. We obtain the edge points coordinates of ground truth image and down-sample the size to the size of original probability map and employ binary cross-entropy loss as the loss function. First-P-Net is trained using the Adam optimizer [64] with a batch size $b_{1}=1$ and a learning rate $\lambda_{1}=1\times 10^{-5}$. The training epochs for both datasets are 10 epochs, and the proposed model takes 2.1 and 4.3 h on average to train an epoch on Sunnybrook 2009 dataset and ACDC 2017 dataset, respectively. The difference in training time is due to the inequality training samples in the two datasets. All the experiments are run on a single NVIDIA GeForce GTX 1080 Ti card.

In the training process, 21,000 state transitions are collected to build an experience replay buffer before Next-P-Net’s learning. When the replay buffer is full, the newest transition will replace the oldest transition, and the evaluation network starts to update its parameters every time step using the Adam optimizer with a batch size $b_{2}=256$ and a learning rate $\lambda_{2}=1\times 10^{-4}$. The parameters of the evaluation network are assigned to the target network every 2000 iterations. In nskipneighborhoods, *n* is set to 5. The maximum step for an agent to find the contour is set to 300 steps empirically. After 100 steps, if the distance between the next point and the first point is less than 40, *n* changes into 3 to obtain more dense and accurate segmentation results. When the distance is less than 20, this episode is ended and breaks down early to stop the agent from repeatedly finding the image contour. Otherwise, the agent keeps operating until it spends 300 steps. After network convergence, the agent took only 100 to 150 steps to find the closed contour (around 0.9 s per image). For exploration, ϵ greedy policy with ϵ set to 0.2 is employed in both training and testing process. The discount factor γ is set to 0.9.

### 4.3. Performance

**Figure 7:**
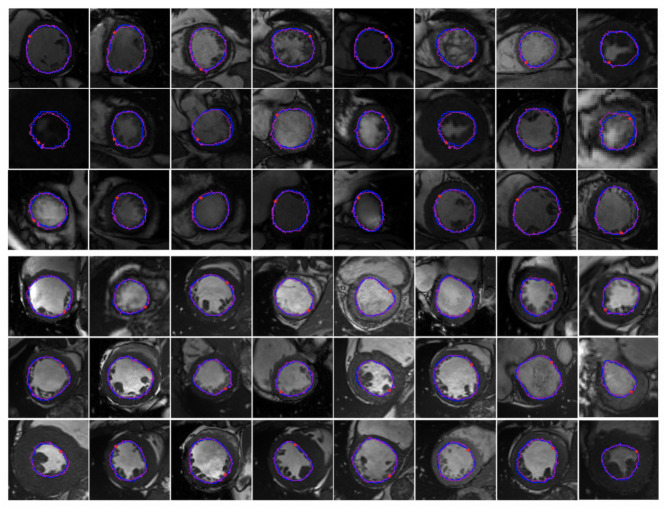
Examples of segmentation outcomes. The first three rows are the segmentation performances on Sunnybrook 2009 testing dataset and the last three rows are the segmentation performances on ACDC 2017 testing dataset. The ground truth (GT) boundary is plotted in blue and the magenta dots are the points found by Next-P-Net. The red pentagram represents the first edge point found by First-P-Net.

**Figure 8:**
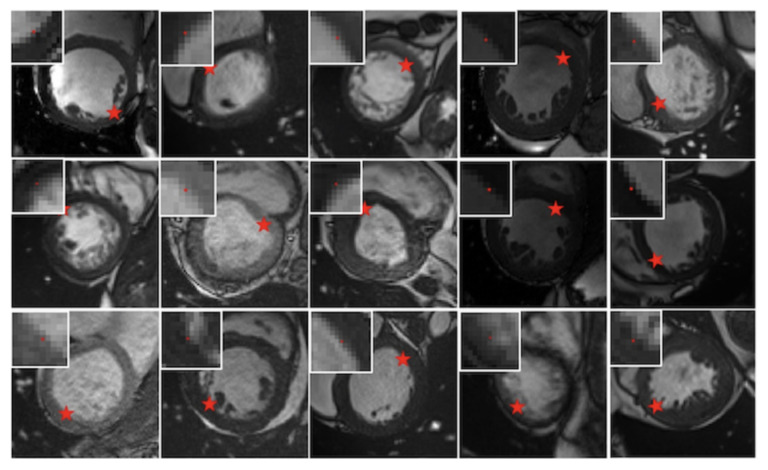
Examples of the first edge point found by First-P-Net on ACDC 2017 testing dataset. The red pentagram represents the first edge point and the small image on the upper left or upper right corner is the partial enlargement of the first point.

Figure 7 shows some segmentation outcomes on Sunnybrook 2009 testing dataset and ACDC 2017 testing dataset. The first three rows are the segmentation performances on Sunnybrook 2009 testing dataset and the last three rows are the segmentation performances on ACDC 2017 testing dataset. No post-processing steps are adopted for better boundary smoothness. Some examples of the first edge point found by First-P-Net on the ACDC 2017 testing dataset are depicted in Figure 8. The red pentagram represents the first edge point and the small image on the upper left or upper right corner is the partial enlargement of the first point. From Figure 8, we can see that the first point found by First-P-Net is very accurate. The first point locates nearby the image edge, which provides the DRL agent a suitable initial state. From Figure 7, the LV contours segmented by the proposed system are consistent with the ground truth contours, which shows the validity of the proposed model. In some LV images, there exists depressions caused by trabeculations and papillary muscles since typically, they have a similar intensity as the myocardium in magnetic resonance imaging. However, the DRL agent can still make sensible actions. The reason is that First-P-Net is trained with ground truth images and the probability map it produced has encoded the information that the LV is a round object. Therefore, the probability map, as one of the concatenated layers of state, can provide the DQN agent prior object shape knowledge and prevent the agent from getting stuck in depressions.

**Figure 9:**
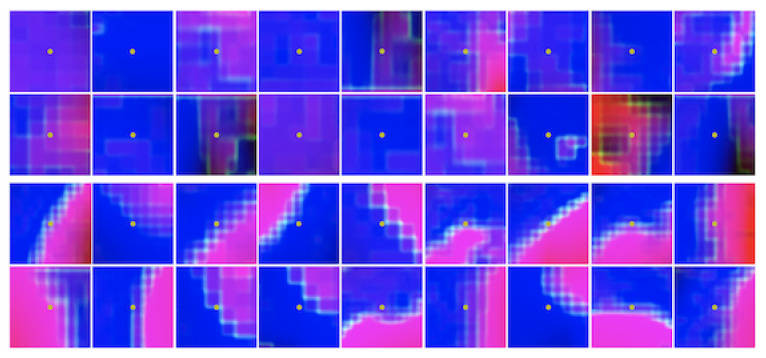
States with different Q-values on ACDC 2017 testing Dataset. The first two rows show some images centered on the yellow point with low Q-value, while the last two rows show some images centered on the yellow point with high Q-value.

The Q-value function Q(s,a) evaluates the expected cumulative reward starting from state *s*, taking action *a*. Based on the definition of state, if an edge point is centered on the cropped image, it is a promising state and the corresponding Q-value of this state tends to be high. Conversely, if there is no edge point on the cropped image, no matter what action the agent makes, this state owns lower Q-value. Figure 9 illustrates some states with different Q-values. Comparing the states of high Q-values and low Q-values, we can see that high Q-value states have obvious boundary pixels appearing in the center of the images while the center points of low Q-value states do not locate any boundary pixels.

### 4.4. Ablation Study

State, action and reward function are the key elements in the design of a DRL system. To analyze the effect of these elements in the proposed model, we employed different forms of state and reward to train the model. Experiments were carried out on ACDC 2017 dataset by changing only the corresponding components while keeping other parts intact.

**State**: In addition to the original grayscale layer, the defined state concatenates four additional layers, which are Sobel layer (**S**), cropped probability map layer (**C**), global probability map layer (**G**) and past points’ map layer (**P**) (**Experiment 0: SCGP**). The state contains more detailed information than the original grayscale image. To verify the effectiveness of state, we trained Next-P-Net by exploiting other simpler state representations for comparison. One representation of the state consists of three layers by omitting two probability map layers (**Experiment 1: SP**). Hence, the layers of state defined in Experiment 1 are grayscale layer, Sobel layer and past points map layer. Another state is composed of four layers by omitting past points’ map layer (**Experiment 2: SCG**).

**Figure 10:**
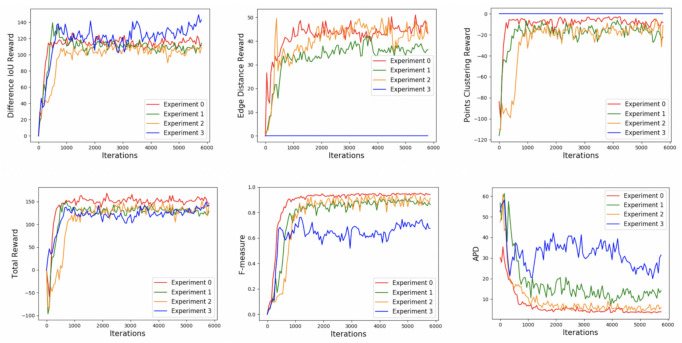
The changes in three separate reward values, total reward value, *F-measure* accuracy and *APD* accuracy according to the learning iterations in the training process on ACDC 2017 Dataset.

The changes in rewards and assessment criteria according to the learning iterations in the training process are plotted in Figure 10. In this figure, one iteration involves the whole training process inside an image. *Average perpendicular distance* (*APD*), *Precision*, *Recall* and *F-measure* (or *DICE*/ *Jaccard* index) are computed to quantitatively evaluate LV boundaries. *APD* measures the distance between the segmented contour and the GT contour, computed by averaging over all segmented contour points. A high value implies that the two contours do not match closely. *F-measure* evaluates the trade-off between *Precision* and *Recall*. From these six graphs, we can see that the upward trends in three separate rewards and the total reward are obvious, implying that our model is learning. Comparing the curves of Experiment 0–2, the total reward and *F-measure* achieved by the proposed state are higher than those achieved by the other experimental states in most training iterations. Besides, the curves of Experiment 0 are more stable than other experiments curves. Comparisons of Experiment 0–2 on ACDC 2017 testing dataset are reported in Table 1. Numbers in bold represent the best results. From this table, we observe that all the assessment criteria of Experiment 0 are better than that of Experiment 1 and Experiment 2. The average *F-measure* of either deleting past points map or two probability map layers on the training dataset is around 0.88. Adding the past points map and probability map together achieved the average *F-measure* of 0.93, showing an increase of 5% compared to using only one of them. Thus, it can verify that both past points map and probability map provide useful image information, and the proposed representation of the state is more comprehensive than using only one of them.

**Figure 11:**
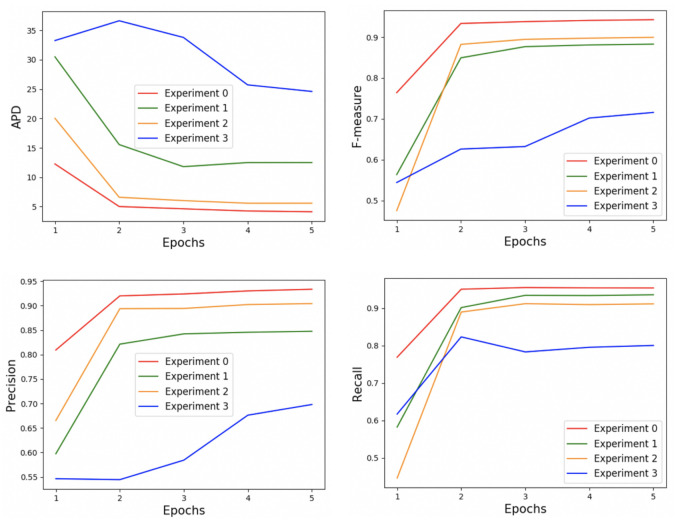
The changes in *APD*, *F-measure*, *Precision* and *Recall* according to the learning epochs in the training process on ACDC 2017 dataset.

**Reward**: The reward function is designed as the sum of difference *IoU* reward (**Rd**), edge distance reward (**Re**) and points clustering reward (**Rp**). To validate the positive effects of the edge distance reward and points clustering reward, we barely employed the difference *IoU* reward as the final immediate reward to train Next-P-Net (**Experiment 3: Rd**) while the state is composed of five layers the same as Experiment 0. From Figure 10, comparing the curves of Experiment 0 and Experiment 3, the difference *IoU* reward sees an increase when discarding edge distance reward and points clustering reward. However, the total reward and average *F-measure* in both training process and testing process decreases. Utilizing the difference *IoU* reward as the final immediate reward only achieved 0.69 *F-measure* on the testing dataset as reported in the last row of Table 1, showing a dramatic decrease of 33% compared to employing the sum of difference *IoU* reward, edge distance reward and points clustering reward. We can conclude that adding edge distance reward and points clustering reward provides a more overall evaluation to the action made by the DQN agent. Figure 11 illustrates the changes in assessment criteria according to the learning epochs in the training process on ACDC 2017 dataset.

### 4.5. Comparison with Other Methods

To verify the effectiveness of the proposed model in LV segmentation, we conducted comparison experiments on ACDC 2017 dataset [62] and Sunnybrook 2009 dataset [63]. To the best of our knowledge, left ventricle image segmentation approaches based on RL are rare [55,56,57]. Wang et al. [55,56] introduced the context-specific segmentation concept and developed a general segmentation framework using reinforcement learning for LV segmentation, but they carried out the experiments on private dataset, achieving 0.895 *F-measure* on healthy LV data and 0.834 *F-measure* on diseased LV data. Mortazi et al. [57] developed a network optimization search algorithm based on policy gradient to perform LV segmentation. In order to conduct a comprehensive experiment, we compare the proposed model with deep learning baselines. In medical image segmentation tasks, popular convolutional neural network architectures include fully convolutional network (FCN) [26], U-Net [2] and their variants, e.g., UNet++ [21], Attention U-Net [22], FCN2 [27], the combination of DeepLab and U-Net [31]. Accuracy comparisons with these deep learning methods are listed in Table 2. Numbers in bold represent the best results. In the experiments, all networks are trained from scratch. From this table, we observe that Attention U-Net and U-Net++ give better segmentation results on both ACDC 2017 dataset and Sunnybrook 2009 dataset. Although in these two public datasets, the segmentation accuracy achieved by the proposed model is not as high as U-Net architecture, it still achieved acceptable LV segmentation performances.

### 4.6. Performance on Small Datasets

In order to discuss the performance of different approaches on small datasets, experiments were held out on 15 subjects randomly selected from [62]. We randomly divided 15 subjects into 3 subsets (Set 1, Set 2, Set 3) and each set contains 5 subjects for 3-fold cross validation. The three subsets contain 82, 86 and 91 images, respectively. In experiments, a single subset is retained as the validation data for testing the model, and the remaining 2 subsets are used as training data. All the models on the three small datasets were trained separately and trained from scratch. In the experiments, we found that the segmentation accuracy achieved by the proposed model increases very rapidly in the beginning. After only 5 epochs, the model has converged and achieved higher segmentation accuracy than FCN and U-Net architecture. The segmentation results of different networks on the small datasets are reported in Table 3. Numbers in bold represent the best results. It achieves the highest *Precision*, *Recall* and *F-measure* accuracy on the small dataset. On the other hand, as the number of training samples decreases, we can see that the segmentation performances of UNet++ and Attention U-Net have deteriorated compared to vanilla U-Net.

The proposed model can efficiently exploit the information inside the training images as this model can be trained with only a few interactions and rapid convergence. However, it requires higher computation loading since the DQN agent needs to find edge points step by step. For each image, it takes 100–150 steps to find the closed contour. For each step, the agent needs to make an action, interact with the environment, obtain the reward, update its parameters through back-propagation, and go to the next state. The computation load of segmenting a single image for the DQN agent is heavier than that for traditional deep learning methods, and this results in a longer time when running an epoch.

## 5. Conclusions

In this paper, unlike the conventional deep learning-based segmentation methods that gradually adjust the segmentation probability of each pixel, we propose a novel edge-based image segmentation model using DRL, and train a DRL agent to delineate the outline of the left ventricle. This agent tries to imitate the behavior of a human observer by finding an initial point on the edge of left ventricle, and then gradually positioning other edge points. This model contains two neural networks: First-P-Net and Next-P-Net. The goal of the First-P-Net is to find the first edge point of the object and generate a probability map of the edge point position. After that, Next-P-Net iteratively locates the coordinates of the next edge point and obtains a closed and accurate segmentation result. Based on deep learning benchmarks of ACDC 2017 dataset and Sunnybrook 2009 dataset, the performance of the proposed model is better than previous reinforcement learning methods and has comparable performance of deep learning baselines. Compared with deep learning methods, the proposed model can also be trained on small datasets with fast convergence and achieves higher *F-measure* accuracy.

However, there are some weaknesses in the proposed model. For instance, once the agent finds the edge of the wrong direction and deviates far from the truth, it is difficult to return in the correct way. More appropriate exploration policy or combining global information should be considered to improve the stability of the DRL agent in the feature work. In addition, we only utilize a probability map to provide prior knowledge of the object shape. In order to better understand the object to be segmented, more high-level information is expected to be added to the state representation. For instance, the feature map before the probability map in the First-P-Net contains richer global and edge information than the probability map. We may skip-connect this feature map with the feature map outputted by Next-P-Net and train the two networks simultaneously. Informative state representation enables the agent to make more sensible actions and achieve better segmentation performance. Besides, the hyper-parameter *n* in *n skip neighborhoods* is fixed in the proposed method. An adaptive step *n* is more flexible to design for the action space in the future.

## Figures and Tables

**Table 1:** Experiments on ACDC 2017 dataset: different state definitions and reward definitions. Numbers in bold represent the best results.

Exp	State	Reward	Difference IoU Reward	Edge Distance Reward	Points Clusting Reward	Total Reward	*APD*	*Precision*	*Recall*	*F-Measure*
0	SCGP	RdReRp	110.9860	44.8845	−3.9499	149.9206	**4.1757**	**0.9383**	**0.9500**	**0.9428**
1	SP	RdReRp	98.6766	35.1291	−4.9411	128.8646	11.6372	0.8580	0.9181	0.8808
2	SCG	RdReRp	96.2734	38.4739	−13.5838	121.1635	6.1673	0.8824	0.9004	0.8825
3	SCGP	Rd	124.3672	0	0	124.3672	24.4755	0.6723	0.8043	0.6997

**Table 2:** Accuracy comparison on ACDC 2017 dataset and Sunnybrook 2009 dataset. Numbers in bold represent the best results.

Dataset	Model	Method		*APD*	*Precision*	*Recall*	*F-Measure*
		DL	RL				
	FCN-8s [26]	✓		4.8724	0.9340	0.9329	0.9326
	U-Net [2]	✓		4.3779	0.9395	0.9401	0.9388
	UNet++ [21]	✓		4.0660	**0.9429**	0.9420	0.9418
ACDC	AttenU-Net [22]	✓		**3.8900**	0.9357	**0.9589**	0.9465
2017 [62]	FCN2 [27]	✓		6.865	-	-	0.94
	DeepLab+U-Net [31]	✓		-	-	-	**0.9502**
	Policy Gradient [57]		✓	8.9	-	-	0.928
	The proposed		✓	4.1757	0.9383	0.9500	0.9428
	FCN-8s [26]	✓		5.4146	0.9418	0.9193	0.9292
	U-Net [2]	✓		5.4541	0.9475	0.9141	0.9295
Sunnybrook	UNet++ [21]	✓		5.2907	0.9242	0.9409	0.9317
2009 [63]	AttenU-Net [22]	✓		**5.1936**	**0.9490**	0.9238	**0.9351**
	The proposed		✓	5.7185	0.9155	**0.9431**	0.9270

**Table 3:** Accuracy comparison with deep learning baselines on the small dataset randomly extracted from ACDC 2017 dataset (3-fold cross validation). Numbers in bold represent the best results.

Train Set	Test Set	Model	*APD*	*Precision*	*Recall*	*F-Measure*
		FCN-8s [26]	8.5395	**0.9147**	0.8513	0.8796
		U-Net [2]	**7.0136**	0.8707	0.8979	0.8828
1 + 2	3	UNet++ [21]	8.4232	0.8217	0.8839	0.8504
		AttenU-Net [22]	7.4199	0.8348	0.9085	0.8689
		The proposed	8.9178	0.8770	**0.9281**	**0.8983**
		FCN-8s [26]	6.5695	0.9307	0.8846	0.9055
		U-Net [2]	5.3193	0.9059	0.9402	0.9216
1 + 3	2	UNet++ [21]	5.8975	0.9153	0.9266	0.9197
		AttenU-Net [22]	5.1295	0.9094	0.9284	0.9175
		The proposed	**4.8773**	**0.9334**	**0.9416**	**0.9357**
		FCN-8s [26]	6.1855	**0.9421**	0.8717	0.9047
		U-Net [2]	**5.4322**	0.9287	0.9079	0.9167
2 + 3	1	UNet++ [21]	6.5104	0.9053	0.8846	0.8931
		AttenU-Net [22]	5.5275	0.9171	0.9111	0.9126
		The proposed	6.4510	0.9041	**0.9385**	**0.9181**
		FCN-8s [26]	7.0982	**0.9292**	0.8692	0.8966
		U-Net [2]	**5.9217**	0.9018	0.9153	0.9070
average result		UNet++ [21]	6.9437	0.8808	0.8984	0.8877
		AttenU-Net [22]	6.0256	0.8871	0.9160	0.8997
		The proposed	6.7481	0.9048	**0.9360**	**0.9173**

## Data Availability

Data available in a publicly accessible repository. The data presented in this study are openly available at [https://acdc.creatis.insa-lyon.fr/description/databases.html, accessed on 10 September 2017] and [https://www.cardiacatlas.org/studies/sunnybrook-cardiac-data/, accessed on 7 September 2009].

## References

[1] Rundo L., Militello C., Vitabile S., Casarino C., Russo G., Midiri M., Gilardi M.C. (2016). Combining split-and-merge and multi-seed region growing algorithms for uterine fibroid segmentation in MRgFUS treatments. Med. Biol. Eng. Comput..

[2] Ronneberger O., Fischer P., Brox T. U-net: Convolutional networks for biomedical image segmentation. Proceedings of the International Conference on Medical Image Computing and Computer-Assisted Intervention.

[3] Noh H., Hong S., Han B. Learning deconvolution network for semantic segmentation. Proceedings of the IEEE International Conference on Computer Vision.

[4] He K., Gkioxari G., Dollar P., Girshick R. Mask r-cnn. Proceedings of the IEEE International Conference on Computer Vision.

[5] Zhao H., Shi J., Qi X., Wang X., Jia J. Pyramid scene parsing network. Proceedings of the IEEE Conference on Computer Vision and Pattern Recognition.

[6] Sutton R.S., Barto A.G. (2018). Reinforcement Learning: An Introduction.

[7] Li Y. Deep Reinforcement Learning. https://arxiv.org/abs/1810.06339.

[8] Mnih V., Kavukcuoglu K., Silver D., Rusu A.A., Veness J., Bellemare M.G., Graves A., Riedmiller M., Fidjeland A.K., Ostrovski G. (2015). Human-level control through deep reinforcement learning. Nature.

[9] Hasselt H.V., Guez A., Silver D. Deep reinforcement learning with double Q-learning. Proceedings of the Thirtieth AAAI Conference on Artificial Intelligence.

[10] Wang Z., Schaul T., Hessel M., Hasselt H.V., Lanctot M., Freitas N.D. Dueling Network Architectures for Deep Reinforcement Learning. https://arxiv.org/abs/1511.06581.

[11] Hausknecht M., Stone P. Deep Recurrent q-Learning for Partially Observable Mdps. https://arxiv.org/abs/1507.06527.

[12] Schaul T., Quan J., Antonoglou I., Silver D. Prioritized Experience Replay. https://arxiv.org/abs/1511.05952.

[13] Caicedo J.C., Lazebnik S. Active object localization with deep reinforcement learning. Proceedings of the IEEE International Conference on Computer Vision.

[14] Bellver M., GiroiNieto X., Marques F., Torres J. Hierarchical Object Detection with Deep Reinforcement Learning. https://arxiv.org/abs/1611.03718.

[15] Codari M., Pepe A., Mistelbauer G., Mastrodicasa D., Walters S., Willemink M.J., Fleischmann D. Deep Reinforcement Learning for Localization of the Aortic Annulus in Patients with Aortic Dissection. Proceedings of the International Workshop on Thoracic Image Analysis.

[16] Ren L., Lu J., Wang Z., Tian Q., Zhou J. Collaborative deep reinforcement learning for multi-object tracking. Proceedings of the European Conference on Computer Vision.

[17] Sallab A.E., Abdou M., Pero E., Yogamani S. (2017). Deep reinforcement learning framework for autonomous driving. Electron. Imaging.

[18] Minaee S., Boykov Y., Porikli F., Plaza A., Kehtarnavaz N., Terzopoulos D. Image Segmentation Using Deep Learning: A Survey. https://arxiv.org/abs/2001.05566.

[19] Chen C., Qin C., Qiu H., Tarroni G., Duan J., Bai W., Rueckert D. (2020). Deep learning for cardiac image segmentation: A review. Front. Cardiovasc. Med..

[20] Litjens G., Ciompi F., Wolterink J.M., de Vos B.D., Leiner T., Teuwen J., Išgum I. (2019). State-of-the-art deep learning in cardiovascular image analysis. JACC Cardiovasc. Imaging.

[21] Zhou Z., Siddiquee M.M.R., Tajbakhsh N., Liang J. Unet++: A nested u-net architecture for medical image segmentation. Proceedings of the Deep Learning in Medical Image Analysis and Multimodal Learning for Clinical Decision Support.

[22] Schlemper J., Oktay O., Schaap M., Heinrich M., Kainz B., Glocker B., Rueckert D. (2019). Attention gated networks: Learning to leverage salient regions in medical images. Med. Image Anal..

[23] Xie L., Song Y., Chen Q. (2020). Automatic left ventricle segmentation in short-axis MRI using deep convolutional neural networks and central-line guided level set approach. Comput. Biol. Med..

[24] Kallenberg M., Petersen K., Nielsen M., Ng A.Y., Diao P., Igel C., Vachon C.M., Holland K., Winkel R.R., Karssemeijer N. (2016). Unsupervised deep learning applied to breast density segmentation and mammographic risk scoring. IEEE Trans. Med. Imaging.

[25] Wang G., Li W., Zuluaga M.A., Pratt R., Patel P.A., Aertsen M., Doel T., David A.L., Deprest J., Ourselin S. (2018). Interactive medical image segmentation using deep learning with image-specific fine tuning. IEEE Trans. Med. Imaging.

[26] Long J., Shelhamer E., Darrell T. Fully convolutional networks for semantic segmentation. Proceedings of the IEEE Conference on Computer Vision and Pattern Recognition.

[27] Abdeltawab H., Khalifa F., Taher F., Alghamdi N.S., Ghazal M., Beache G., Mohamede T., Keyntona R., El-Baz A. (2020). A deep learning-based approach for automatic segmentation and quantification of the left ventricle from cardiac cine MR images. Comput. Med. Imaging Graph..

[28] Liu L., Cheng J., Quan Q., Wu F.X., Wang Y.P., Wang J. (2020). A survey on U-shaped networks in medical image segmentations. Neurocomputing.

[29] Rundo L., Han C., Nagano Y., Zhang J., Hataya R., Militello C., Tangherloni A., Nobile M.S., Ferretti C., Besozzi D. (2019). USE-Net: Incorporating Squeeze-and-Excitation blocks into U-Net for prostate zonal segmentation of multi-institutional MRI datasets. Neurocomputing.

[30] Hu J., Shen L., Sun G. Squeeze-and-excitation networks. Proceedings of the IEEE Conference on Computer Vision and Pattern Recognition.

[31] Galea R.R., Diosan L., Andreica A., Popa L., Manole S., Bálint Z. (2021). Region-of-Interest-Based Cardiac Image Segmentation with Deep Learning. Appl. Sci..

[32] Chen L.C., Zhu Y., Papandreou G., Schroff F., Adam H. Encoder-decoder with atrous separable convolution for semantic image segmentation. Proceedings of the European Conference on Computer Vision.

[33] Militello C., Rundo L., Toia P., Conti V., Russo G., Filorizzo C., Ludovico L.G., Massimo M., Vitabile S. (2019). A semi-automatic approach for epicardial adipose tissue segmentation and quantification on cardiac CT scans. Comput. Biol. Med..

[34] Commandeur F., Goeller M., Razipour A., Cadet S., Hell M.M., Kwiecinski J., Chang H., Marwan M., Achenbach S., Berman B.S. (2019). Fully automated CT quantification of epicardial adipose tissue by deep learning: A multicenter study. Radiol. Artif. Intell..

[35] Moreno R.A., Rebelo D.S.M.F., Carvalho T., Assuncao A.N., Dantas R.N., Val R.D., Marin A.S., Bordignom A., Nomura C.H., Gutierrez M.A. A combined deep-learning approach to fully automatic left ventricle segmentation in cardiac magnetic resonance imaging. Proceedings of the Medical Imaging 2019: Biomedical Applications in Molecular, Structural, and Functional Imaging.

[36] Romaguera L.V., Romero F.P., Costa C.F.F., Costa M.G.F. Left ventricle segmentation in cardiac MRI images using fully convolutional neural networks. Proceedings of the Medical Imaging 2017: Computer-Aided Diagnosis.

[37] Nasr-Esfahani M., Mohrekesh M., Akbari M., Soroushmehr S.R., Nasr-Esfahani E., Karimi N., Samavi S., Najarian K. Left ventricle segmentation in cardiac MR images using fully convolutional network. Proceedings of the 2018 40th Annual International Conference of the IEEE Engineering in Medicine and Biology Society.

[38] Avendi M.R., Kheradvar A., Jafarkhani H. (2016). A combined deep-learning and deformable-model approach to fully automatic segmentation of the left ventricle in cardiac MRI. Med. Image Anal..

[39] Ngo T.A., Lu Z., Carneiro G. (2017). Combining deep learning and level set for the automated segmentation of the left ventricle of the heart from cardiac cine magnetic resonance. Med. Image Anal..

[40] Rupprecht C., Huaroc E., Baust M., Navab N. Deep Active Contours. https://arxiv.org/abs/1607.05074.

[41] Shokri M., Tizhoosh H.R. Using reinforcement learning for image thresholding. Proceedings of the CCECE 2003-Canadian Conference on Electrical and Computer Engineering, Toward a Caring and Humane Technology.

[42] Song G., Myeong H., Lee K.M. Seednet: Automatic seed generation with deep reinforcement learning for robust interactive segmentation. Proceedings of the IEEE Conference on Computer Vision and Pattern Recognition.

[43] Grady L. (2006). Random walks for image segmentation. IEEE Trans. Pattern Anal. Mach. Intell..

[44] Han J., Yang L., Zhang D., Chang X., Liang X. Reinforcement cutting-agent learning for video object segmentation. Proceedings of the IEEE Conference on Computer Vision and Pattern Recognition.

[45] Jegou S., Drozdzal M., Vazquez D., Romero A., Bengio Y. The one hundred layers tiramisu: Fully convolutional densenets for semantic segmentation. Proceedings of the IEEE Conference on Computer Vision and Pattern Recognition Workshops.

[46] Castrejon L., Kundu K., Urtasun R., Fidler S. Annotating object instances with a polygon-rnn. Proceedings of the IEEE Conference on Computer Vision and Pattern Recognition.

[47] Acuna D., Ling H., Kar A., Fidler S. Efficient interactive annotation of segmentation datasets with polygon-rnn++. Proceedings of the IEEE Conference on Computer Vision and Pattern Recognition.

[48] Chitsaz M., Seng W.C. Medical image segmentation by using reinforcement learning agent. Proceedings of the 2009 International Conference on Digital Image Processing.

[49] Chitsaz M., Woo C.S. (2011). Software agent with reinforcement learning approach for medical image segmentation. J. Comput. Sci. Technol..

[50] Tian Z., Si X., Zheng Y., Chen Z., Li X. (2020). Multi-step medical image segmentation based on reinforcement learning. J. Ambient. Intell. Humaniz. Comput..

[51] Dong N., Kampffmeyer M., Liang X., Wang Z., Dai W., Xing E. Reinforced auto-zoom net: Towards accurate and fast breast cancer segmentation in whole-slide images. Proceedings of the Deep Learning in Medical Image Analysis and Multimodal Learning for Clinical Decision Support.

[52] Sahba F., Tizhoosh H.R., Salama M.M. A reinforcement learning framework for medical image segmentation. Proceedings of the 2006 IEEE International Joint Conference on Neural Network Proceedings.

[53] Sahba F., Tizhoosh H.R., Salama M.M. (2008). Application of reinforcement learning for segmentation of transrectal ultrasound images. BMC Med. Imaging.

[54] Liao X., Li W., Xu Q., Wang X., Jin B., Zhang X., Zhang Y., Wang Y. Iteratively-Refined Interactive 3D Medical Image Segmentation with Multi-Agent Reinforcement Learning. Proceedings of the IEEE/CVF Conference on Computer Vision and Pattern Recognition.

[55] Wang L., Merrifield R., Yang G.Z. Reinforcement learning for context aware segmentation. Proceedings of the International Conference on Medical Image Computing and Computer-Assisted Intervention.

[56] Wang L., Lekadir K., Lee S.L., Merrifield R., Yang G.Z. (2013). A general framework for context-specific image segmentation using reinforcement learning. IEEE Trans. Med. Imaging.

[57] Mortazi A., Bagci U. Automatically designing CNN architectures for medical image segmentation. Proceedings of the International Workshop on Machine Learning in Medical Imaging.

[58] Mahmud M., Kaiser M.S., Hussain A., Vassanelli S. (2018). Applications of deep learning and reinforcement learning to biological data. IEEE Trans. Neural Netw. Learn. Syst..

[59] Girshick R. Fast r-cnn. Proceedings of the IEEE International Conference on Computer Vision.

[60] He K., Zhang X., Ren S., Sun J. Deep Residual Learning for Image Recognition. Proceedings of the IEEE Conference on Computer Vision and Pattern Recognition.

[61] Ioffe S., Szegedy C. Batch normalization: Accelerating deep network training by reducing internal covariate shift. Proceedings of the International Conference on Machine Learning.

[62] Bernard O., Lale A., Zotti C., Cervenansky F., Yang X., Heng P.A., Jodoin P.M. (2018). Deep Learning Techniques for Automatic MRI Cardiac Multi-structures Segmentation and Diagnosis: Is the Problem Solved?. IEEE Trans. Med. Imag..

[63] Radau P., Lu Y., Connelly K., Paul G., Dick A., Wright G. Evaluation framework for algorithms segmenting short axis cardiac MRI. Midas J. Card. Left Ventricle Segm. Chall..

[64] Kingma D.P., Ba J. Adam: A method for stochastic optimization. Proceedings of the International Conference for Learning Representations.

